# Supplementary figures and images for: Potential of phenothiazines to synergistically block calmodulin and reactivate PP2A in cancer cells
Source: PLoS One. 2022 May 26;17(5):e0268635. doi: 10.1371/journal.pone.0268635 (PMC9135253; doi:10.1371/journal.pone.0268635)

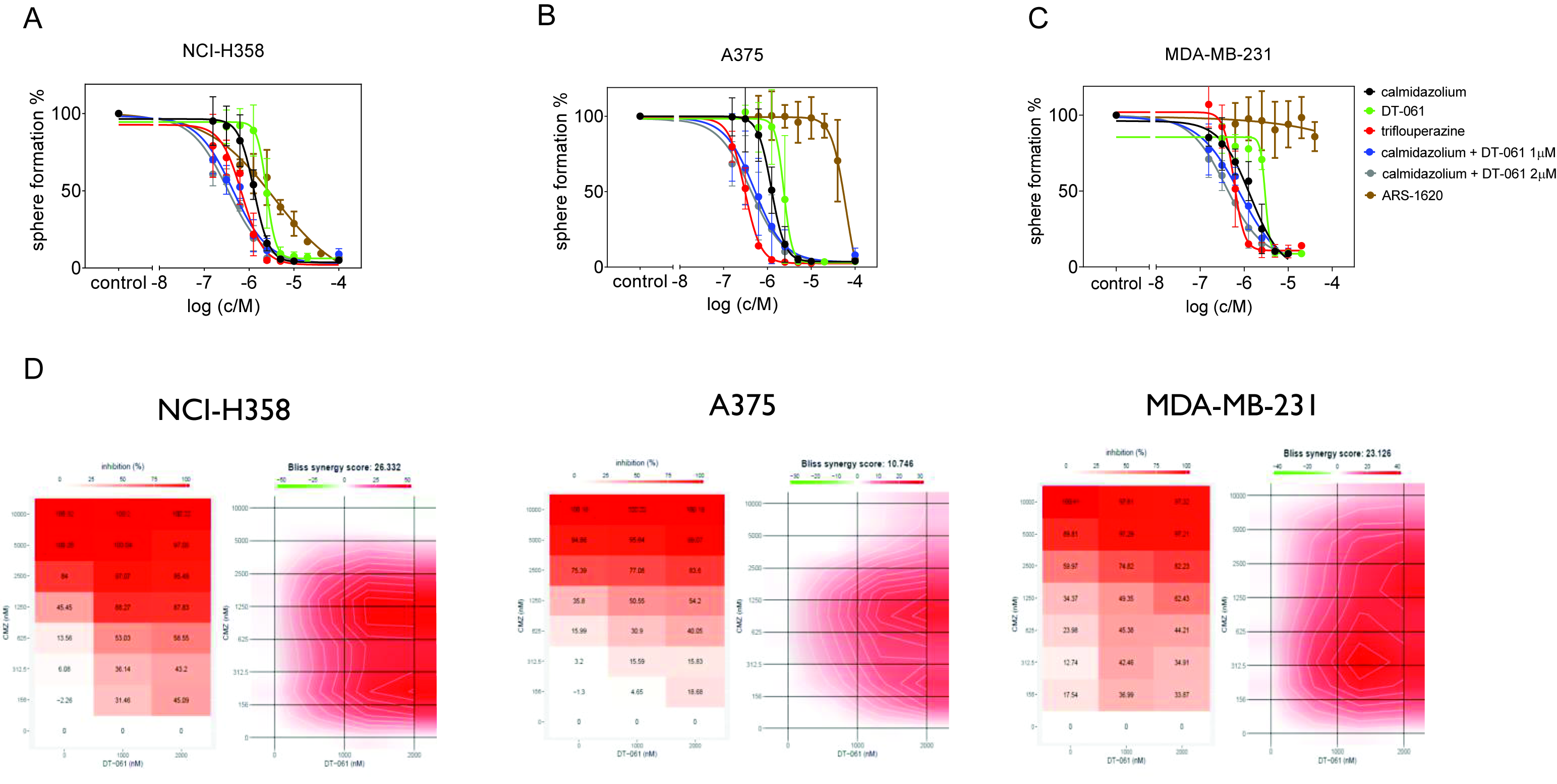

Supplement: S1 Fig — (A-C) Dose response curves for indicated inhibitors in Ras pathway mutant cell lines NCI-H358 (A), A375 (B) and MDA-MB-231 (C). Compounds were tested as either single agents at concentration ranges of 0.2 μM– 10 μM (calmidazolium), 0.6 μM– 40 μM (DT-061), 0.2 μM– 40 μM (trifluoperazine) or 0.6 μM– 40 μM (ARS-1620), or in combination at a full dose response range of 0.2 μM– 10 μM for calmidazolium with 1 or 2 μM of DT-061 added to all test conditions. Data represent mean values ± SD, n = 2. The data were fit into log (inhibitor) vs variable response (four parameters) function in the Prism (GraphPad) to obtain the dose response curves. The actual curve fitting for Bliss calculation was done on the SynergyFinder website (https://synergyfinder.fimm.fi/). (D) Representative Bliss synergism heatmaps for combinatorial effects of calmidazolium and DT-061 in Ras mutant cancer cell lines. Heatmaps with positive scores (shaded red) indicate synergistic drug interaction whereas, heatmaps with negative scores (shaded green) indicates antagonistic drug interaction. (TIF) [file pone.0268635.s001.tif]

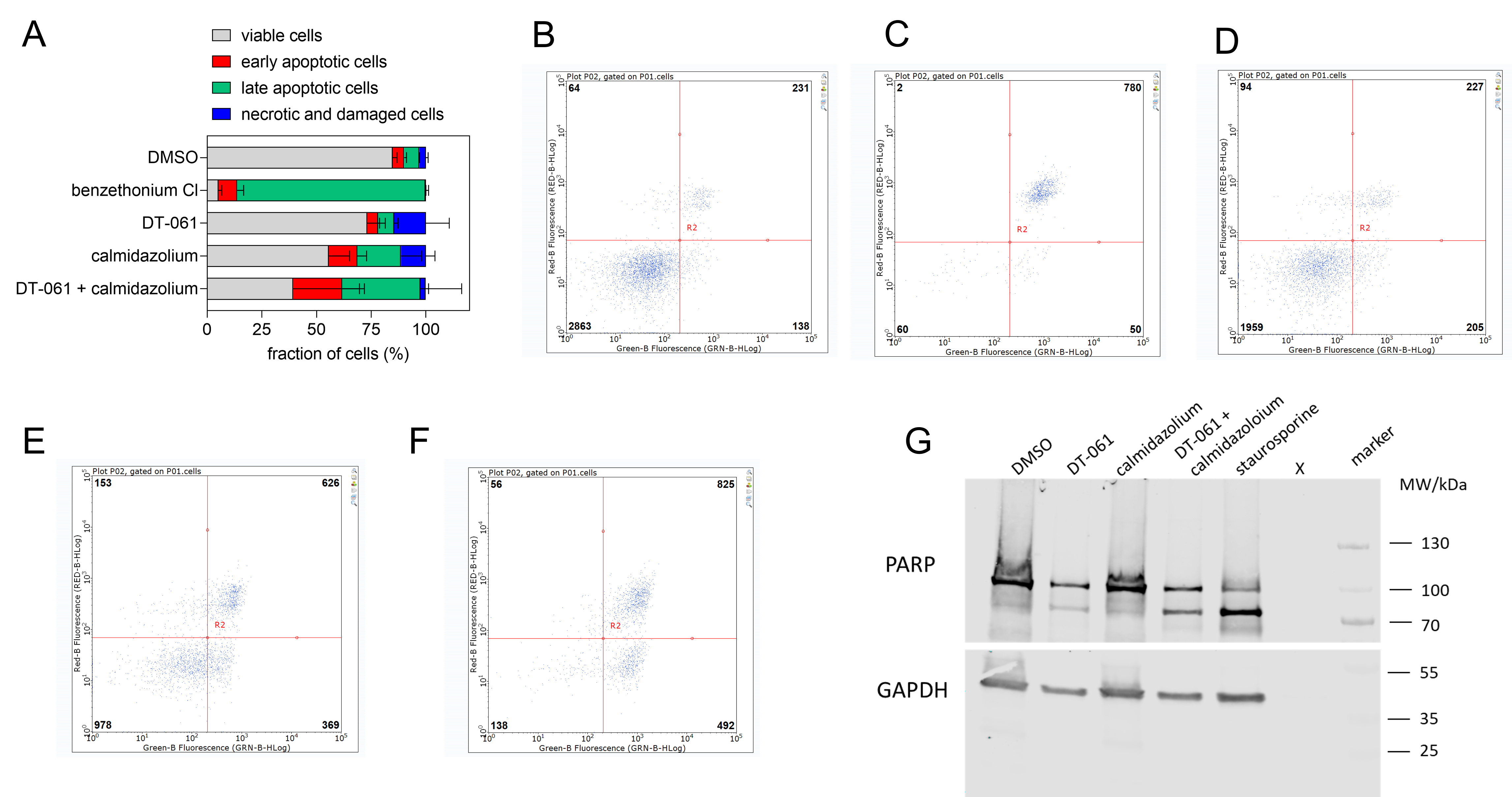

Supplement: S2 Fig — (A) Percentage of viable cells (non-stained), early apoptotic cells (Annexin V-FITC stained), late apoptotic cells (both annexin V-FITC and 7AAD stained) and necrotic and damaged cells (7AAD stained), representing each quadrant of a dot plot is presented. Data represent mean ± SD of two independent biological repeats. (B-F) Representative dot plots of DMSO (B), benzethonium chloride (C), DT-061 (D), calmidazolium (E), and combination of DT-061 and calmidazolium (F) from one biological repeat. (G) Uncropped Western blot membrane presented in Fig 1F. After the transfer the membrane was cut between 70 and 55 kDa and the top and bottom blots were probed with anti-PARP and anti-GAPDH antibody, respectively. X and marker indicate non-related sample and PageRuler (ThermoScientific) protein ladder, respectively. (TIF) [file pone.0268635.s002.tif]

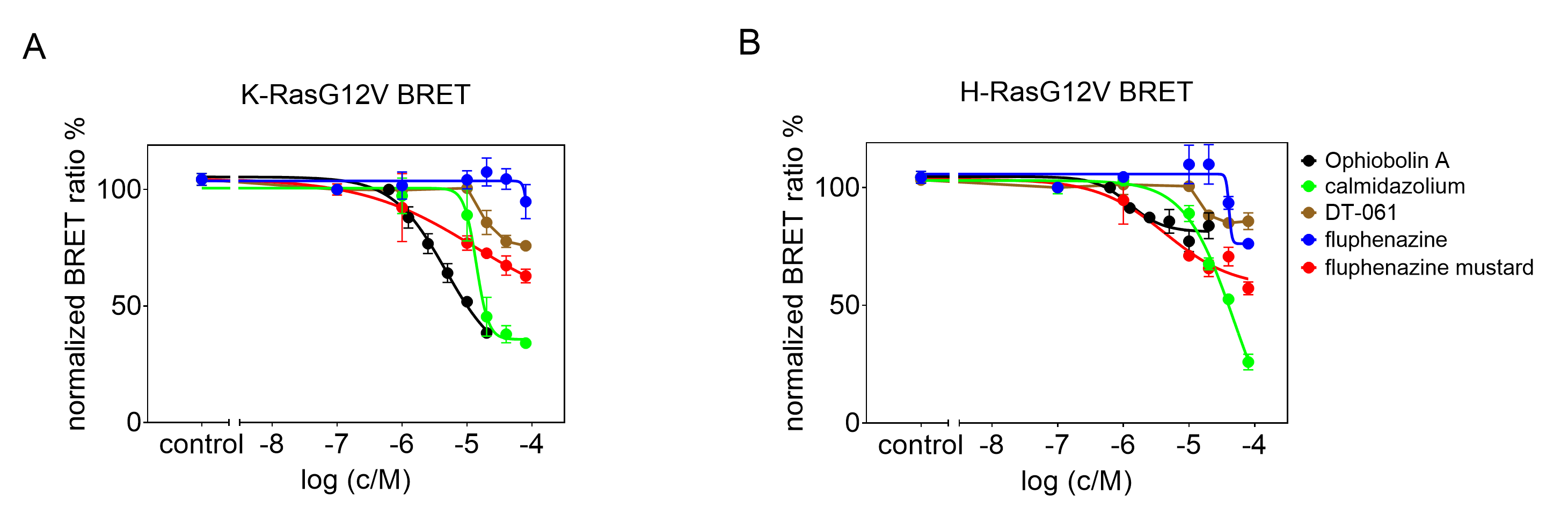

Supplement: S3 Fig — (A, B) Dose response curves of fluphenazine and fluphenazine mustard (0.1–80 μM), calmidazolium (0.1–80 μM), DT-061 (0.1–80 μM) and OphA (0.3–20 μM) using the K-RasG12V (A) or H-RasG12V (B) nanoclustering-BRET assays in HEK-293 EBNA cells. The A/D plasmid ratio was 4/1. Data represent mean values ± SD, n ≥ 3. The data were fit into log (inhibitor) vs variable response (four parameters) function of Prism (GraphPad) to obtain the dose response curves. (TIF) [file pone.0268635.s003.tif]

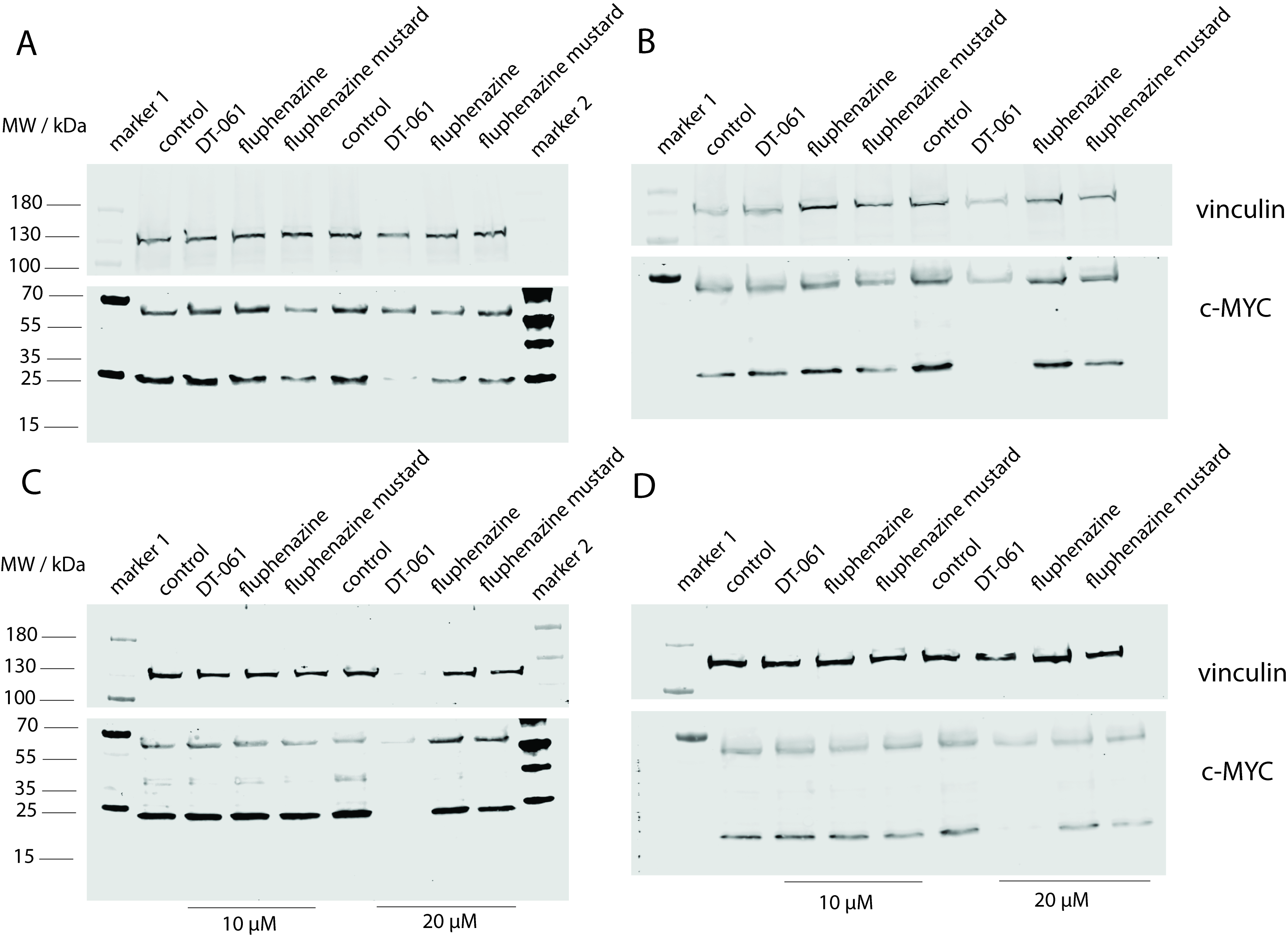

Supplement: S4 Fig — (A-D) Measurement of c-MYC expression after compound treatment in MDA-MB-231 cells from four independent biological repeats. Uncropped Western blot membranes of all biological repeats are presented. After the transfer the membrane was cut between 100 and 70 kDa and the top and bottom blots were probed with anti-vinculin and anti-c-MYC antibody, respectively. Control indicates 0.1% (v/v) and 0.2% (v/v) DMSO treatments. Marker 1 and marker 2 indicate PageRuler (ThermoScientific) and unstained Precision Plus (Bio-Rad) protein ladders. Note that the anti-c-MYC antibody (#5605, Cell signaling) strongly detected an unknown protein at 25 kDa in MDA-MB-231 cell lysate compared to c-MYC, which was detected in between 55 and 70 kDa. (TIF) [file pone.0268635.s004.tif]

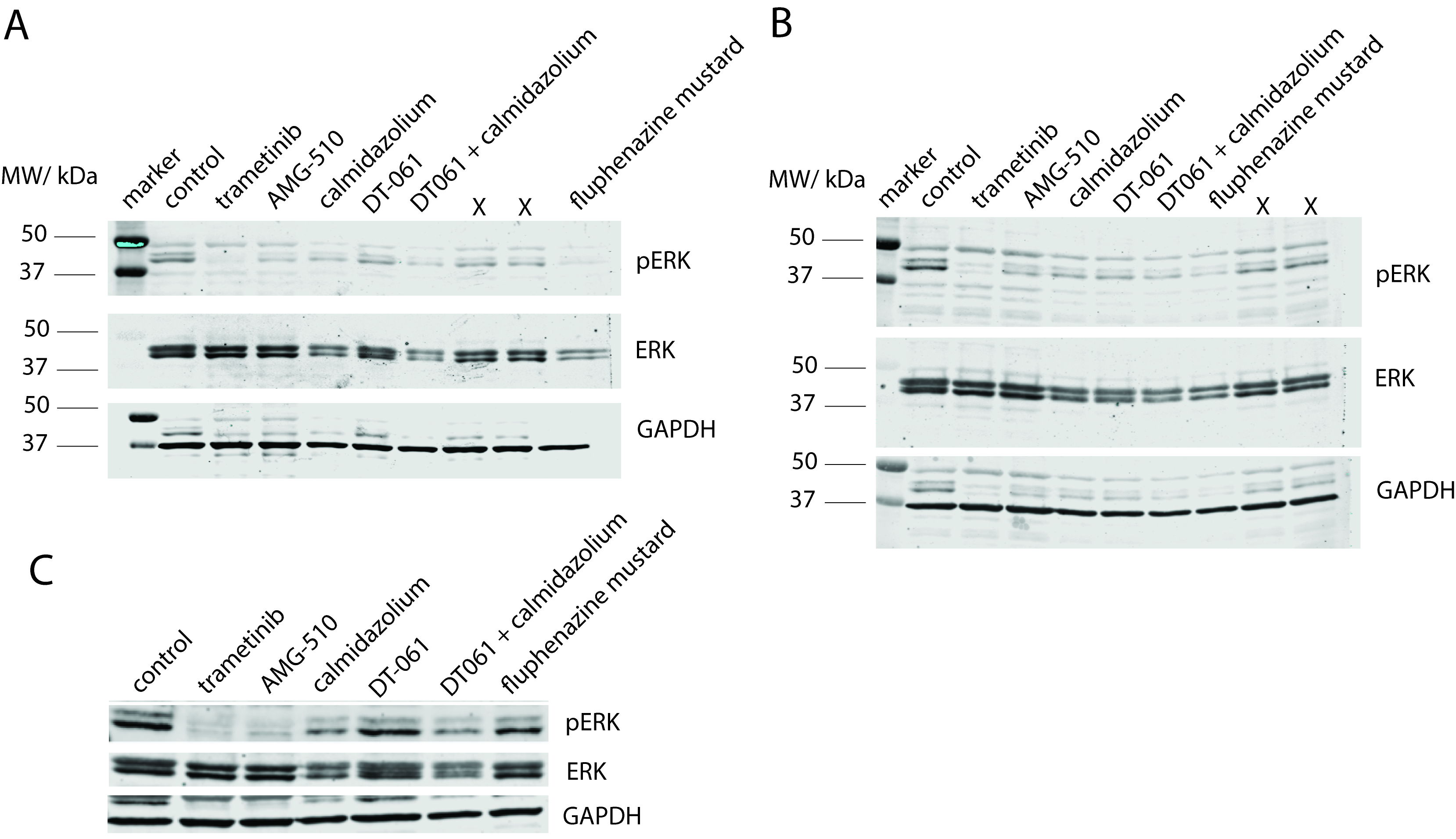

Supplement: S5 Fig — MAPK signaling output measurement in MIA PaCa-2 cells upon treatment with control compounds trametinib (1 μM) and AMG-510 (1 μM), single agent treatment with calmidazolium (20 μM) and DT-061 (20 μM) or in combination (DT-061 10 μM + CMZ 10 μM) as well as fluphenazine mustard (20 μM). (A, B) Uncropped Western blot membranes of two biological repeats and (C) a cropped Western blot membrane from a third biological repeat are presented. The membrane was first probed for ERK and pERK levels simultaneously. After developing the membrane using secondary antibodies, the same membrane was then again probed for GAPDH. X and marker indicate non-related sample and unstained Precision Plus (Bio-Rad) protein ladder, respectively. (TIF) [file pone.0268635.s005.tif]

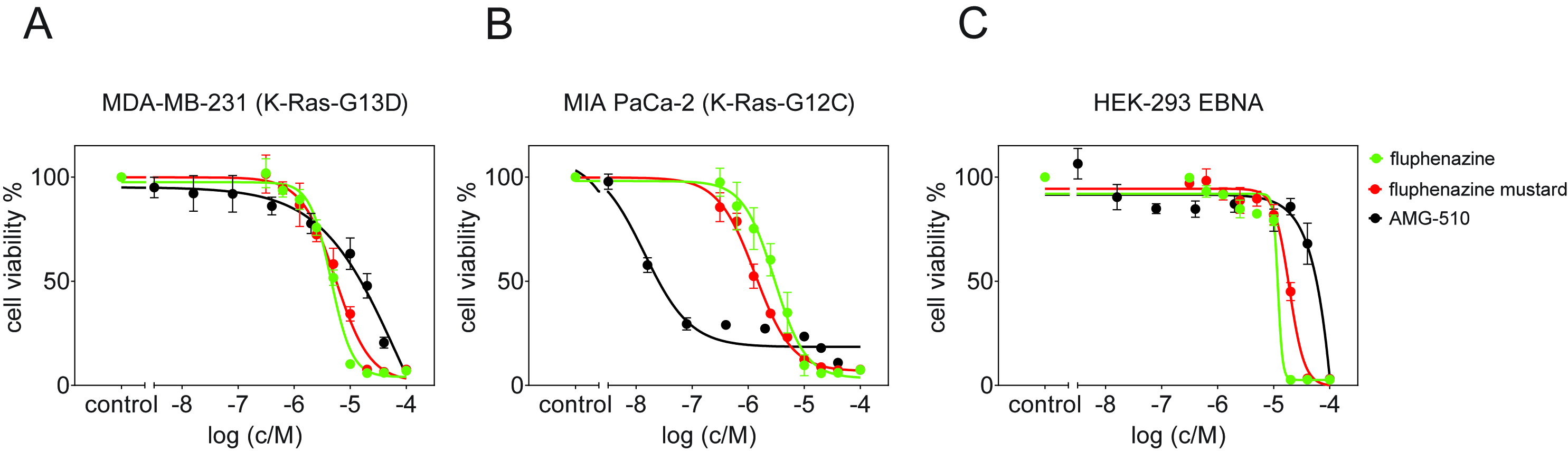

Supplement: S6 Fig — Comparison of effects of compounds on proliferation of MDA-MB-231 (A), MIA PaCa-2 (B) and HEK-293 EBNA (C) cells grown in 2D monolayers. Cells were treated with compound at concentration ranges of 0.6 μM– 80 μM (fluphenazine and fluphenazine mustard) and 0.003 μM– 40 μM (AMG-510). Data represent mean values ± SD, n = 3. The data were fit to log (inhibitor) vs response–variable slope (four parameters) equation of Prism (GraphPad) software. The actual curve fitting for DSS3 calculation was done on the DSS platform Breeze. (TIF) [file pone.0268635.s006.tif]
