# Supplementary material for: Potential of phenothiazines to synergistically block calmodulin and reactivate PP2A in cancer cells
Source: PLoS One. 2022 May 26;17(5):e0268635. doi: 10.1371/journal.pone.0268635 (PMC9135253; doi:10.1371/journal.pone.0268635)
Supplement: Attachment — Submitted filename: Manoharan_Response to Reviewers.docx [file pone.0268635.s008.docx]

**Point by point response to revision on manuscript ID PONE-D-21-23656 entitled “Potential of phenothiazines to synergistically block calmodulin and reactivate PP2A in cancer cells”**

**Reviewer #1:**

**The paper is an interesting investigation of the ability of phenothiazine derivatives to synergistically act on two cancer associated targets, calmodulin and the tumor suppressor protein phosphatase. The methodology was well chosen, and the findings are sound. I recommend its publication after some minor revisions, as follows.**

**(1) The Abstract should be more accurate in presenting the compounds investigated in the paper.**

**(2) The structure of the investigated PTZs should be included**

**(3) A Conclusions section should be included.**

**Responses 1:**

We thank the reviewer for their positive comments. We improved the manuscript based on the suggestions of the reviewer:

1. The abstract was improved and mentions the exact compounds employed in the study.
2. The structures of all investigated compounds are now provided in **Scheme 1**.
3. A conclusion section has been added.

**Reviewer #2:**

**Phenothiazines (PPZs) are widely used as antipsychotic drugs because of their inhibitory activity toward monoamine neurotransmitter receptors. Some of the PPZs bind directly to PP2A holoenzyme and enhance its activity. Recently, PPZ-derived small-molecule activators of PP2A reported exerting anti-cancer effects. Moreover, some of the PPZs have inhibitory activity toward Calmodulin (CaM). CaM and PP2A modulate the Ras pathway. Therefore, targeting both CaM and PP2A simultaneously is a promising strategy for Ras-mutated cancers. However, it remains unclear whether PPZ-derivatives retain both CaM inhibitory and PP2A activatory functions. The authors show here that PPZ-derivative fluphenazine mustard (Flu-M) has anti-CaM activity and claimed that PTZ integrates the synergistic activities of CaM inhibition and PP2A activation within one compound. Their story is potentially interesting, but there is a definite lack of data.**

**(1)** **The major problem is that there is no data that Flu-M has PP2A inhibitory effect. The authors need to measure PP2A activity or show that Flu-M treatment promotes the constitution of the PP2A holoenzyme.**

**Response 2-1:**

We thank the reviewer for the comments, which helped us to improve the manuscript. The activation of PP2A by small molecules such as DT-061 leads to the degradation of c-MYC (Farrington et. al., 2020, JBC), thus by measuring the expression of c-MYC, the PP2A modulatory activity of the compounds could be evaluated. In **Fig 4A**, the PP2A modulatory activity of fluphenazine and fluphenazine mustard is reported along with DT-061, confirming that all compounds can reduce c-MYC expression levels at certain concentration ranges.

**(2)** **Figure 1A: The authors present results from the DRIVE database and argue that the survival of K-Ras mutant and BRAF mutant cells is dependent on the PP2A and CaM genes (P11, L250). However, it is only MDA-MB-231 cells that both PP2A and CaM are in "red: a highly dependent". Authors should state more correctly.**

**Response 2-2:**

We thank the reviewer for pointing this out and are now stating on P14, L274: ‘Of note, only the MDA-MB-231 breast cancer cell line showed a dependency on *KRAS, CALM3* as well as *PPP2CA*.’

**(3)** **Figure 1B: In relation to Figure 1A, why does calmidazolium treatment induce cell death in A375 to the same extent as in other cells, even though A375 survival is CaM-independent? The same is true for the effects of DT-061.**

**Response 2-3:**

We thank the reviewer for this interesting observation. There are several explanations, one could suggest, however, the major point may be that knockdown of a gene and loss of its protein product (all active sites lost) may have a very different effect than (incompletely) inhibiting a specific site on a protein (one site lost). Moreover, a compound may have other (unknown) related targets. Different (transcriptional) compensatory responses may result.

Moreover, the ATARiS data for the 3 CaM genes, which encode identical proteins, show different dependencies on the 3 CaM genes. This may add a cautionary note to the interpretation of the ATARiS data, as factors such as specific expression level or compensatory responses may have to be factored in. ATARiS data are therefore highly valuable to indicate genetic dependencies, but cannot be equated with inhibitor effects.

**Minor point.**

**(1) P3, L47: treatment e.g. of bipolar > treatment of e.g. bipolar**

Corrected as suggested.

**(2) P12, L252: alpha subunit of the PP2A enzyme > alpha isoform of the PP2A enzyme**

Corrected as suggested.

**Reviewer #3:** **In this manuscript "Potential of phenothiazines to synergistically block calmodulin and reactivate PP2A in cancer cells" the authors present interesting data related to the potential utility of using calmodulin inhibitors and PP2A activators to synergistically modulate downstream pathways and drive cancer cell specific death. Overall, the work is well done and represent an interesting conceptual advance in the field. There are several weaknesses that should be addressed prior to this manuscript being suitable for publication.**

**(1)** **Additional assays should be performed to determine effects and mechanisms of cell death - for example colony formation assays with the drugs alone and in combination would be of interest. Additionally, FACS analysis with Annexin and PI staining would be of interest; western blotting for markers of apoptosis such as cleaved caspase 3 and PARP would also strengthen the conclusions from Figure 1**

**Response 3-1:**

We thank the reviewer for the interesting and encouraging comments.

We have carried out suggested flow cytometry and apoptosis marker experiments.

The effect of inhibitors and combinations on cell apoptosis was analyzed by staining treated cells with annexin V and 7-AAD, and analyze marker intensity per cell using flow cytometry/FACS (**Fig 1D; S2 Fig**). These data show that the combination of DT-061 (PP2A-activator) and calmidazolium (CaM inhibitor) have a higher activity than the individual compounds alone.

The caspase-3 activity of compounds is now reported using CaspaseGlo assay (**Fig 1E**), similar to data published in literature (Kauko et al., 2018, Sci Trans Med).

In addition, the effect of compounds on PARP cleavage using Western blotting is reported (**Fig 1F**), confirming that the combination induced more PARP cleavage than compounds alone.

**(2) Was the BRET assay in Figure 2B performed with DT-061?**

**Response 3-2:**

We have now updated **Fig 2B and C** showing the effect of DT-061 in the K-Ras/CaM BRET assay. In line with the in vitro experiments in **Fig 2A**, this compound had no effect.

**(3) In Figure 4 the MIAPACA2 pancreatic cancer cell line was used for the studies - what was the rationale for the selection of this cell line; the cell lines used in Figure 1 should be tested for effects of pERK and tERK and more doses and time points should be used to quantitate drugs effects on MAPK signaling - additionally, the authors should look at pMEK and tMEK expression in these experiments.**

**Response 3-3:**

The MiaPaCa cell line was used in order to compare the effect of Flu-M with a clinically approved Ras pathway inhibitor AMG-510.

We apologize, but within this limited study, with one co-author having left the lab, our experimental capacity was insufficient to perform the requested MAPK-signalling investigation on all cell lines studied. The focus in this last dataset is on a higher activity of Flu-M (**Fig 4C,D**), and the comparison with the clinical compound AMG-510 in cell culture and an assessment of the selective toxicity against cancer cell lines (**Fig 4E**).

In the abstract, we now clearly state that this is a limited study.

Nevertheless, we now report the PP2A modulating activity of Flu and Flu-M by assessing the c-MYC expression level, as this is attributed to the PP2A reactivation by SMAPs, such as DT-061 (**Fig 4A**,**B**).
