# Supplementary material for: Potential of phenothiazines to synergistically block calmodulin and reactivate PP2A in cancer cells
Source: PLoS One. 2022 May 26;17(5):e0268635. doi: 10.1371/journal.pone.0268635 (PMC9135253; doi:10.1371/journal.pone.0268635)
Supplement: Attachment — Submitted filename: Manoharan_Response to Reviwers.docx [file pone.0268635.s009.docx]

**Point by point response to revision on manuscript ID PONE-D-21-23656R2 entitled “Potential of phenothiazines to synergistically block calmodulin and reactivate PP2A in cancer cells”**

**Reviewer #2:**

**At the initial review, I mentioned the following point.**

**(1) The major problem is that there is no data that Flu-M has PP2A inhibitory effect. The authors need to measure PP2A activity or show that Flu-M treatment promotes the constitution of the PP2A holoenzyme.**

**The authors responded to this by measuring the c-Myc protein level as an alternative to measuring PP2A activity. Unfortunately, c-Myc is not an indicator of PP2A activity. PP2A-B56 complex dephosphorylates Ser62 of c-Myc and destabilizing it, on the other hand, PP2A-B55 complex dephosphorylates Thr58 of c-Myc and stabilizing it. Whether Flu-M has a PP2A activating effect is the crux of this paper. Therefore, as I first pointed out, the authors should either measure PP2A activity or show that the formation of the PP2A complex is promoted.**

**Response:**

We apologize that the reviewer feels we still have not satisfactorily addressed their concern.

1) However, as the reviewer will know, production and reconstitution of the PP2A holoenzyme is technically demanding and even PP2A-focused research groups, such as e.g. from Drs. Narla (Farrington et al., 2020; PMID: 31822503) and Westermarck (Kauko et al., 2018; PMID: 30021885) employ the c-MYC destabilization assay as a surrogate to measure PP2A activation.

The former group established in the above publication that the anti-tumor activity of DT-061 depends on the Ser62-dephosphorylation dependent c-MYC degradation.

Furthermore, the way we introduce c-MYC degradation as surrogate assay, and how we conclude our findings, reflects the limitations of our assay: L. 416- *Both Flu and Flu-M decreased the c-MYC level in MDA-MB-231 cells (****Fig 4A,B and S4 Fig****) comparable to that of DT-061, supporting their PP2A activation.*

2) For the first revision, we unsuccessfully attempted to obtain the PP2A-A/ B56alpha Nano-BRET construct described in Leonard et al., 2020 (PMID: 32315618). However, it was not possible to obtain these constructs from the community, and again such an assay would have had limitations by focusing only on one B subunit.

3) Our major point is that two properties of phenothiazine derivatives, the retention of their CaM inhibitory and PP2A activating properties, could be important to improve them as anti-cancer agents. The CaM inhibition has previously been neglected in the development of PP2A activators. We believe that our admittedly limited study still provides data for thought in ongoing PP2A activator development projects. This conclusion does not only rest on fluphenazine mustard but is likewise supported by fluphenazine and the combination of DT-061 and calmidazolium (**Figure 4 A,B**).

We have clarified the limitations pointed out by the reviewer with the following statement in the adapted manuscript and hope that the reviewer would accept this, given that in vitro reconstitution studies are out of reach for us:

L. 460- *One clear limitation of our study is that we have not directly examined PP2A activation by PTZ, for example by reconstitution of the holoenzyme, but instead employed surrogate measures such as c-MYC degradation.*
